# Functional *Toll-Like Receptor (TLR)2* polymorphisms in the susceptibility to inflammatory bowel disease

**DOI:** 10.1371/journal.pone.0175180

**Published:** 2017-04-07

**Authors:** Helga Paula Török, Victor Bellon, Astrid Konrad, Martin Lacher, Laurian Tonenchi, Matthias Siebeck, Stephan Brand, Enrico Narciso De Toni

**Affiliations:** 1 Department of Medicine II, Ludwig-Maximilians-University, Munich, Germany; 2 MINES ParisTech, PSL-Research University, CBIO-Centre for Computational Biology, Fontainebleau, France; 3 Institut Curie, Paris,France; 4 INSERM U900, Paris, France; 5 Department of Pediatric Surgery, University of Leipzig, Leipzig, Germany; 6 Department of General, Visceral, Vascular and Transplantation Surgery, Ludwig-Maximilians-University, Munich, Germany; University Tuebingen, GERMANY

## Abstract

**Background:**

The recent genome-wide association studies (GWAS) in inflammatory bowel disease (IBD) suggest significant genetic overlap with complex mycobacterial diseases like tuberculosis or leprosy. *TLR* variants have previously been linked to susceptibility for mycobacterial diseases. Here we investigated the contribution to IBD risk of two *TLR2* polymorphisms, the low-prevalence variant Arg753Gln and the GT_*n*_ microsatellite repeat polymorphism in intron 2. We studied association with disease, possible correlations with phenotype and gene-gene interactions.

**Methodology/Principal findings:**

We conducted a large study in 843 patients with Crohn’s disease, 426 patients with ulcerative colitis and 805 healthy, unrelated controls, all of European origin. Overall, the frequency for carriers of shorter GT_*n*_ repeats in intron 2 of the *TLR2* gene, which have previously been associated with low *TLR2* expression and high IL-10 production, was slightly elevated in Crohn’s disease and ulcerative colitis compared to healthy controls (16.0% resp. 16.7% vs. 12.8%). The highest frequency of short GT_*n*_ carriers was noted among IBD patients on anti TNF-alpha therapy. However, none of these differences was significant in the multivariate analysis. The Arg753Gln polymorphism showed no association with any clinical subtype of IBD, including extensive colitis, for which such an association was previously described. We found no association with specific phenotypic disease subgroups. Also, epistasis analysis revealed no significant interactions between the two *TLR2* variants and confirmed IBD susceptibility genes.

**Conclusions:**

The two functional relevant polymorphisms in *TLR2*, the GT_*n*_ microsatellite repeat polymorphism in intron 2 and the Arg753Gln variant do not seem to play a role in the susceptibility to Crohn’s disease or ulcerative colitis.

## Introduction

Among complex diseases some of the most notable progress has been made in the genetic characterisation of the inflammatory bowel diseases (IBD) Crohn’s disease (CD) and ulcerative colitis (UC). Large-scale genome-wide association studies (GWAS) and extensive meta-analyses facilitated by international collaborative research groups led to the identification of 200 IBD-associated loci, of which 163 are associated with both diseases, 37 are CD specific and 27 are UC specific [1, 2]. Besides shared loci for IBD and other immune-mediated disorders such as psoriasis and ankylosing spondylitis, one of the key findings of the latest meta-analyses of the GWAS and ImmunoChip data is a considerable overlap between susceptibility for IBD and mycobacterial infection: six of the eight known autosomal genes linked to Mendelian susceptibility to mycobacterial disease are located within IBD loci; as regards complex mycobacterial disease, seven CD susceptibility genes overlap with leprosy susceptibility genes [1].

Toll-like receptors are transmembrane proteins usually expressed by antigen presenting cells; they act as receptors of the innate immune system by recognizing specific pathogen-associated molecular patterns with subsequent activation of immune responses. In the digestive system TLRs can recognize invading microbes in the intestinal barrier and activate immune responses. However, an over-activation of these receptors may lead to chronic intestinal inflammation. Because of the ability of TLRs to recognise particular molecular patterns of diverse microorganisms, their contribution to disease susceptibility has been studied for various mycobacterial diseases as well as for IBD. Several observations report an association of *TLR* variants with mycobacterial disease [3]. Regarding IBD, variants in *TLR4* have consistently been associated with CD and UC [4] and interactions between a *TLR9* variant and replicated CD susceptibility loci seem to modulate disease susceptibility [5]. Functional polymorphisms in *TLR2*, which result in impaired response to bacterial lipoproteins or influence promotor activity [6, 7], have also been repeatedly associated with susceptibility to mycobacterial disease [8] and other infectious conditions [9] as well as common conditions such as atopic sensitization in the general population [10].

Genetic factors identified by GWAS explain only a modest part of disease variance in IBD (about 13.6% for CD and 7.5% for UC) [1]. This implies that other factors such as environmental exposure, epigenetics but also genetic factors not captured by GWAS contribute substantially to disease pathogenesis. Risk loci with a minor allele frequency >5% in the general population and an odds ratio (OR) >1.2 have presumably all been identified in IBD patients with European ancestry. Other genetic risk factors such as rare variants, copy number variations and microsatellite polymorphisms, however, are still expected to be identified. In the present study we examined in a large European population the contribution to susceptibility for IBD of two such variations of the *TLR2* gene, the low-prevalence variant Arg753Gln (rs5743708) and the GT_*n*_ microsatellite repeat polymorphism in intron 2. Of these two, the Arg753Gln variant has been shown to cause impaired mucosal repair because of a deficient ability to induce TFF3 synthesis [11] and has previously been associated with severe (extensive) disease in ulcerative colitis [12]. The GT_*n*_ repeat microsatellite polymorphism in intron 2 of the *TLR2* gene was first described in 2004; the study found high variability in the numbers of GT repeats starting at -100 bp from the ATG and ranging from 12 to 28 repeats [7]. The distribution of allele lengths significantly differs among racial groups, and the number of GT repeats seems to have functional implications. Short GT repeats have been shown to result in higher *TLR2* promotor activity [7, 13], and after stimulation with TLR2 agonists they result in higher production of pro-inflammatory cytokines (TNF-alpha, IL-12 and IL-6) [13] and lower production of anti-inflammatory cytokines (IL-10) [14]. Therefore, it has been speculated, that short GT repeats are much more prone to inflammation than mid-sized repeats, which are most abundant in every race [7]. Similar to CD-associated *NOD2* variants [15], the microsatellite polymorphism has recently been associated with susceptibility to develop spontaneous bacterial peritonitis in cirrhotic patients [16]. Furthermore, both polymorphisms have previously been linked to mycobacterial disease [8, 17–20], which displays an important genetic overlap with IBD, as mentioned above.

## Methods

### Ethics statement

The study was approved by the Ethics committee of the Medical Faculty of the Ludwig-Maximilians-University Munich. Written, informed consent was obtained from all patients prior to the study. Study protocols were based on the ethical principles for medical research involving human subjects of the Helsinki Declaration.

### Study population and IBD phenotype assessment

We recruited a large cohort comprising 2074 individuals of European origin. This population included 1269 patients with IBD (CD, N = 843; UC, N = 426) and 805 healthy, unrelated controls. All participants included in the study were Caucasians. The patients were all recruited at the University Hospital Munich, Germany. The diagnosis of CD or UC was established by conventional clinical, radiological, endoscopic and histopathological criteria [21]. Patients with indeterminate colitis were excluded from the study. The control population comprised ethnically matched, healthy, unrelated blood donors. Demographic data are given in Table 1.

**Table 1:** Demographic characteristics of the study population.

	Crohn’s disease N = 843	Ulcerative colitis N = 426	Controls N = 805
**Gender**			
Male (%)	48.9	50.2	55.5
Female (%)	51.1	49.8	44.5
**Age (y)**			
Mean (SD)	34.7 (14.3)	37.4 (16.0)	45.6 (10.8)
Range	5–79	3–83	18–73
**Body mass index**			
Mean (SD)	23.1 (4.2)	23.8 (4.0)	
Range	13–41	15–41	
**Age at diagnosis (y)**			
Mean (SD)	25.0 (12.4)	27.9 (14.6)	
Range	1–78	1–81	
**Disease duration (y)**			
Mean (SD)	8.5 (8.2)	7.5 (7.1)	
Range	<1–41	<1–38	
**Positive family history of IBD**			
% of participants	19.1	19.0	0.0

Extensive clinical characterization was available for 760 patients with CD and 375 patients with UC. Phenotypic data were collected by analysing patient charts and from a detailed questionnaire completed during an interview at the time of enrolment. The phenotypic classification of CD and UC patients was based on the Montreal classification and included age at diagnosis (A), location (L) and behaviour (B) of disease for CD and disease extension (E) for UC [22]. The phenotypic data for patients with CD and UC are given in Tables 2 and 3, respectively.

**Table 2:** Phenotypic characteristics of patients with Crohn’s disease for whom detailed phenotypic data was available.

Phenotypic subgroups	n (% of subgroup)
**Age at diagnosis (Montreal A, n = 760)**	
A1, below 16 y	188 (24.7)
A2, between 17 and 40 y	484 (63.7)
A3, above 40 y	88 (11.6)
**Location (Montreal L, n = 806)**	
L1, ileal	105 (13.0)
L2, colonic	185 (23.0)
L3, ileocolonic	505 (62.7)
L4, isolated upper disease	11 (1.3)
**Behaviour**^1^ **(Montreal B, p = perianal disease modifier, n = 782)**	
B1, non-stricturing, non-penetrating	208 (26.6)
B1p	21 (2.6)
B2, stricturing	186 (23.8)
B2p	9 (1.2)
B3, penetrating	320 (40.9)
B3p	38 (4.9)
Any stenosis^2^	432 (55.2)
	**n/total analysed (%)**
Extra-intestinal manifestations	225/469 (48.0)
Surgery because of Crohn’s disease^3^	393/774 (50.8)
Use of immunosuppressive agents^4^	356/437 (81.5)
Anti-TNF-alpha therapy	181/526 (34.4)

^1^Disease behaviour was defined according to the Montreal classification. A stricturing disease phenotype was defined as presence of stenosis without penetrating disease. The diagnosis was made surgically, endoscopically or radiologically (MRI enteroclysis).

^2^Presence of stenosis independent of penetrating disease

^3^Only surgery related to problems specific to Crohn’s disease (e.g. fistulectomy, colectomy, ileostomy) was included

^4^Immunosupressive agents included azathioprine, 6-mercaptopurine, 6-thioguanin, MTX and anti-TNF-alpha agents

**Table 3:** Phenotypic characteristics of patients with ulcerative colitis for whom detailed phenotypic data was available.

Phenotypic subgroups	n (% of subgroup)
**Location (Montreal E, n = 375)**	
E1, ulcerative proctitis	43 (11.5)
E2, left sided ulcerative colitis	118 (31.5)
E3, extensive ulcerative colitis	214 (57.1)
	**n/total analysed (%)**
Extra-intestinal manifestations	60/166 (36.1)
Use of immunosuppressive agents	146/192 (76.0)
Anti-TNF-alpha therapy	56/227 (24.7)

### Genotyping

Genomic DNA was isolated from peripheral blood leucocytes with a commercially available kit from Qiagen (Hilden, Germany) according to the manufacturer’s guidelines. Genotyping of the polymorphism Arg753Gln (rs5743708) in the *TLR2* gene was performed by restriction fragment length polymorphism analysis, as previously described [23]. For genotyping of the microsatellite polymorphism in intron 2 of the *TLR2* gene we used polymerase chain reaction (PCR) to amplify a region of 131–163 bp surrounding the GT repeat microsatellite, as previously described [6, 7]. The number of GT repeats was identified by length analysis of the PCR products with an automatic sequencer. The total volume of the PCR mixture was 10 μl; the mixture contained 50 ng of genomic DNA, 1×PCR buffer (Qiagen, Hilden, Germany), 0.2mM of each dNTP (Sigma, Taufkirchen, Germany), 0.25 units of HotStar-Taq^™^DNA polymerase (Qiagen) and 0.25μM each of the two primers 5′FAM-GCATTGCTGAATGTATCAGGGA-3′ (forward, containing the fluorescein marker 6-carboxyfluorescein [FAM]) and 5′-CTTGAGAAATGTTTTCTAGGC-3′ (reverse; TIB MOLBIOL, Berlin, Germany). The final concentration of MgCl2 was 2mM. After an initial denaturation step at 95°C for 15 min, samples were subjected to 35 cycles of denaturation at 94°C for 30 s, annealing at 55°C for 30 s and elongation at 72°C for 30 s. This temperature regimen was followed by a final elongation step at 72°C for 10 min. The resulting fragments were run on an ABI 3700 sequencer. Samples for which genotypes were previously confirmed by sequencing, i.e. (GT)_13_, (GT)_19_, (GT)_23_, and (GT)_24_, were used as “gold standards” and were run in each gel separately.

Genotype information for the GT_*n*_ repeat microsatellite polymorphisms in intron 2 of the *TLR2* gene was already available for 590 of the controls [16]. Genotypic data for the three CD-associated *NOD2* variants (rs2066844 = p.Arg702Trp, rs2066847 = p.Gly908Arg and rs2066847 = p.Leu1007fsX1008) were available from previous studies [5].

### Statistical analysis

Statistical analysis was performed with SPSS software version 14.0 (SPSS Inc, Chicago, IL) and Python. The genotype frequencies for all investigated polymorphisms were tested for consistency with the Hardy-Weinberg equilibrium.

For the case-control analysis, genotypes and allele frequencies were compared by employing χ² over the weights of a logistic regression, with age, sex and the three first components of a multidimensional scaling (MDS) as covariates. Bonferroni correction was applied for multiple comparisons. *P* values < 0.05 were considered significant.

To test the microsatellite size effect we performed a logistic regression test for the different thresholds. We repeated the test on random permutations of the phenotype to study the distribution of *P* values.

The polymorphism information content (PIC) score for the GT_*n*_ repeat microsatellite marker in our study population was calculated by using the online PIC calculator (http://w3.georgikon.hu/pic/english/kezi.aspx).

A classical linkage equilibrium test was performed with a χ² test between the microsatellite and rs5743708 polymorphism.

## Results

### Case-control association study of the GT_*n*_ repeat microsatellite polymorphism and the Arg753Gln (rs5743708) polymorphism in the *TLR2* gene

The distributions of genotypes for both *TLR2* polymorphisms were consistent with Hardy-Weinberg equilibrium.

**Fig 1:**
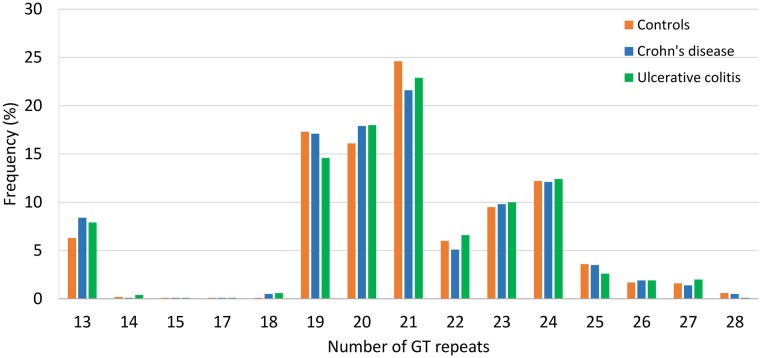
Allele frequencies for the GT_*n*_ repeat microsatellite polymorphism in the study groups.

The number of GT repeats in intron 2 of the *TLR2* gene varied between 13 and 28 in both the disease groups and the healthy controls (see Fig 1). The polymorphic information content for the GT_*n*_ repeat microsatellite polymorphism was 0.833, which can be considered highly informative.

Given the trimodal distribution of GT repeats, we first categorized the alleles into three subclasses, as previously described for the analysis of microsatellite polymorphisms [24]. The lower component with ≤ (GT)_16_ was designated as shorter “S allele”, the middle component between (GT)_17_ and (GT)_22_ as the middle “M allele” and the upper component with ≥ (GT)_23_ as the long “L allele (Table 4). We than analysed the genotype distribution and divided the six genotypes (S/S, S/M, S/L, M/M, M/L, L/L—see S1 Table) according to the presence or absence of the S-allele in genotypes including the S-allele (S-allele carriers) or genotypes without the S-allele (no S-allele carrier). This classification relied on observations from previous studies of the microsatellite polymorphism suggesting the presence of short GT repeats to be dominant over the presence of longer GT repeats [14]. Overall, the frequency of the S-allele and also the genotype frequency for S-allele carriers were slightly higher in patients with CD and UC compared to controls (Table 4). However, after correction for multiple testing, the multivariate analysis including sex, age and the first three components of an MDS as covariates failed to show a significant association of the microsatellite polymorphism with CD or UC.

**Table 4:** Frequencies for the *TLR2* intron 2 microsatellite GT_*n*_ repeats in the study population.

	Crohn’s disease (N = 843)		Ulcerative colitis (N = 426)		IBD (N = 1269)		Controls (N = 805)
	Frequency %	P value, OR [95% CI]	Frequency %	P value, OR [95% CI]	Frequency %	P value, OR [95% CI]	Frequency %
**Allele frequencies**^2^							
S (GT_*n*_, *n*≤16)	8.6	p^3^ = 0.030, 1.34[1.02–1.75]	8.3	n.s.	8.5	p^3^ = 0.026, 1.33[1.03–1.70]	6.5
M (GT_*n*,_ 16<n<22)	62.3	n.s.	62.7	n.s.	62.4		64.3
L (GT_*n*_, *n*≥16)	29.1	n.s	29.0	n.s.	29.1		29.1
**Genotype frequencies**							
S-allele carriers	16.0	n.s.	16.7	n.s.	16.4	p^3^ = 0.031, 1.33[1.03–1.73]	12.8
No S-allle	84.0		83.3		83.6		87.2

^1^The category IBD (inflammatory bowel disease) represents the combined Crohn’s disease (CD) and ulcerative colitis (UC) cohort.

^2^S≤ (GT)_16_, (GT)_17_ < M < (GT)_22_ and L ≥ (GT)_23._ Allelic and genotypic test *P* values and OR (odds ratios) with 95% CI (confidence intervals) are shown for the CD and UC groups compared to controls.

^3^Significant tests (p<0.05) in the univariate analysis, loss of significance after correction for multiple testing.

As previously described in a report on spontaneous bacterial peritonitis in patients with liver cirrhosis [16], we next focused on finding a possible cut-off for the number of GT repeats that could best differentiate between patients and controls. First, we used a 10-fold cross validation for the different cut-offs to classify between controls and CD and UC patients but did not obtain a significant result. Then, we compared the *P* values obtained by logistic regression with the general distribution of *P* values and randomized the phenotype. The best results for both CD and UC were obtained with the cut-off set at 18 GT repeats. We next also used the cut-off 18, i.e. GT_*n*_, *n*≤18 or *n*>18, to test whether the allele and genotype frequencies for the GT_*n*_ repeat microsatellite polymorphism differed significantly between the study groups. The allele frequency for short GT repeats (*n*≤18) was higher in CD patients and the combined IBD group than in controls (9.1% vs. 6.8%). Correspondingly, the frequency of carriers of short GT repeats (i.e. at least one short GT_*n*_, *n*≤18 allele) was higher in the CD, UC and combined IBD groups (17.2%, 17.8% and 17.4%, respectively) than in controls (13.2%). However, also with this cut-off the multivariate analysis found no significant associations of the microsatellite polymorphism after correction for multiple testing (data not shown).

The allelic and genotype distributions of the SNP Arg753Gln (rs5743708) in the *TLR2* gene showed no significant differences between patients with CD or UC and controls. The allele and genotype frequencies and the results of the univariate analysis are shown in Table 5.

**Table 5:** Frequencies for the *TLR2* Arg753Gln (G/A) polymorphism in the study population.

	Crohn’s disease (N = 837)		Ulcerative colitis (N = 401)		IBD^1^ (N = 1238)		Controls (N = 784)
	Frequency %	*P* value, OR[95% CI]	Frequency %	*P* value, OR[95% CI]	Frequency %	*P* value, OR[95% CI]	Frequency %
**Allele frequencies**							
A	3.3	n.s.	3.9	n.s.	3.5	n.s.	3.2
**Genotype frequencies**							
AA	0.3	n.s.	0.0	n.s.	0.2	n.s.	0.1
AG	6.0		7.7		6.6		6.1
GG	93.7		92.3		93.2		93.8

^1^The category IBD (inflammatory bowel disease) represents the combined Crohn’s disease (CD) and ulcerative colitis (UC) cohort. Allelic and genotypic test *P* values and OR (odds ratios) with 95% CI (confidence intervals) have been calculated for the CD, UC and IBD groups compared to controls. No significant associations (p<0.05) resulted in the univariate analysis.

### Genotype-phenotype analysis: No significant association of the *TLR2* polymorphisms with clinical subtypes in CD and UC

We further tested for a specific association of the *TLR2* polymorphisms with clinical subtypes in CD and UC. Such an association has already been described for the Arg753Gln (rs5743708) polymorphism e.g. with extensive colitis in UC [12]. Furthermore, an association of the polymorphisms with a specific clinical subgroup could possibly be responsible for the moderate differences in the allele and genotype distributions observed for the microsatellite polymorphism in the case-control study. Patients were categorised according to their genotype into short allele carriers (at least one S allele) or not short allele carriers (both GT_*n*_ alleles M or L). Similarly, for the Arg753Gln polymorphism carriers of at least one mutated allele (homozygous or heterozygous Arg753Gln carriers) were compared with Arg753Gln wildtype individuals. The results of the subgroup analyses are shown in Tables 6 and 7 for CD and UC, respectively.

**Table 6:** Frequencies of carriers of at least one short (S-allele) for the GT_*n*_ microsatellite polymorphisms in the specific phenotypic subgroups for Crohn’s disease.

*TLR2* microsatellite GT_*n*_ repeat	S- allele carriers / Total (%)	p, OR [95% CI]^1^
**Age at diagnosis (Montreal A, n = 760)**		
A1, below 16 y	31 / 188 (16.5)	n.s.
A2, between 17 and 40 y	81 / 484 (16.7)	n.s.
A3, above 40 y	6 / 88 (6.8)	p = 0.018, 0.37 [0.14–0.90]
**Location (Montreal L, n = 806)**		
L1, ileal	17 / 105 (16.2)	n.s.
L2, colonic	33/ 185 (17.8)	n.s.
L3, ileocolonic	80 / 505 (15.8)	n.s.
L4, isolated upper disease	0 / 11 (0.0)	n.s.
**Behaviour (Montreal B, n = 782)**		
B1, non-stricturing, non-penetrating	34 / 229 (14.9)	n.s.
B2, stricturing	33 / 195 (16.9)	n.s.
B3, penetrating	59/ 358 (16.5)	n.s.
Any stenosis	72 / 432 (16.7)	n.s.
**Extra-intestinal manifestations**	35 / 225 (15.6)	n.s.
**Surgery because of CD**	62/ 393 (15.8)	n.s.
**Use of immunosuppressive agents**	62/ 356 (17.4)	n.s.
Anti-TNF-alpha therapy	34 / 181 (18.8)	n.s.

^1^Allelic and genotypic test *P* values and OR (odds ratios) with 95% CI (confidence intervals) are shown for short S-allele carriers compared to those who were not short allele carriers (both GT_*n*_ alleles M or L) in the specific clinical subgroups. Significant tests (p<0.05) in the univariate analysis are shown as values, not significant tests as shown n.s.

**Table 7:** Frequencies of carriers of at least one short (S-allele) for the intron 2 microsatellite repeat polymorphism or at least one Arg753Gln allele in the *TLR2* gene in the specific phenotypic subgroups for ulcerative colitis.

***TLR2* microsatellite GT**_***n***_ **repeats**	**S-allele carriers / Total (%)**	**p, OR [95% CI]**^1^
**Location (Montreal E, n = 375)**		
E1 (Ulcerative proctitis)	6 / 43 (14.0)	n.s.
E2 (Left sided ulcerative colitis)	21 / 118 (17.8)	n.s.
E3 (extensive ulcerative colitis)	37 / 214 (17.3)	n.s.
**Extra-intestinal manifestations**	14 / 60 (23.0)	p = 0.048, 2.38 [0.95–6.04]
**Use of immunosuppressive agents**	30 / 146 (20.5)	n.s.
Anti-TNF-alpha therapy	12 / 56 (21.4)	n.s.
***TLR2* Arg753Gln**	**Arg753Gln allele carriers / Total (%)**	**P, OR [95% CI]**^2^
**Location (Montreal E, n = 375)**		
E1 (Ulcerative proctitis)	3 / 43 (7.0)	n.s.
E2 (Left-sided ulcerative colitis)	6 / 108 (5.6)	n.s.
E3 (Extensive ulcerative colitis)	16 / 203 (7.9)	n.s.
**Extra-intestinal manifestations**	5 / 54 (9.3)	n.s.
**Use of immunosuppressive agents**	7 / 131 (5.3)	n.s.
Anti-TNF-alpha therapy	4 / 49 (8.2)	n.s.

^1^Allelic and genotypic test *P* values and OR (odds ratios) with 95% CI (confidence intervals) are shown for S-allele carriers compared to those who were not short allele carriers (both GT_*n*_ alleles M or L) in the specific clinical subgroups.

^2^Allelic and genotypic test *P* values and OR (odds ratios) with 95% CI (confidence intervals) are shown for Arg753Gln allele carriers compared to Arg753Gln wildtype individuals in the specific clinical subgroups. Significant tests (p<0.05) in the univariate analysis are shown as values, not significant tests as shown n.s.

As shown in Table 6, the frequency of short GT_*n*_ allele carriers in the group of CD patients with age at diagnosis above 40 years (Montreal A3, 6.8%) was lower than in the groups with age at diagnosis below 16 years (Montreal A1; 16.5%) and between 17 and 40 years (Montreal A2; 16.7%) and lower than in the controls (12.8%). However, only 88 CD patients were included in the subgroup Montreal A3, and the difference was not significant after correction for multiple testing. Similarly, the frequency of carriers of at least one S -allele was higher in CD patients with isolated colonic disease (17.8%) and in CD patients needing immunosuppressive therapy (16.7%), especially anti-TNF-alpha agents (18.8%), than in CD patients with no need for immunosuppressive therapy, but this differences were also not significant after correction for multiple testing.

In patients with ulcerative colitis the frequency of S-allele carriers was also slightly higher in patients needing immunosuppressive therapy, in particular anti-TNF-alpha therapy (21.4%), and in those with extraintestinal disease manifestations (23%), but again these differences were not significant after correction for multiple testing.

Regarding the polymorphism Arg753Gln, we did not find a significantly higher frequency of Arg753Gln carriers in patients with extensive ulcerative colitis (see Table 7), although this has been previously described [12]. Analyses revealed no other significant association with clinical subgroups for CD or UC.

### Gene-gene interactions

On a functional level, the TLR2-mediated response to bacterial peptidoglycan is modulated by NOD2 and this modulation is disturbed in the presence of *NOD2* mutations associated with CD [25, 26, 27]. We tested here for evidence of genetic interactions between the polymorphisms in *TLR2* and disease-associated *NOD2* variants with possible implications for susceptibility to CD. Such interactions have been described for spontaneous bacterial peritonitis in cirrhotic patients, with a significant increase in disease risk in the presence of both disease-associated *NOD2* variants and long GT_*n*_ repeats for the *TLR2* microsatellite polymorphism [16]. The frequency of short (S-allele) carriers for the GT_*n*_ repeat microsatellite polymorphism in *TLR2* was slightly higher among CD patients carrying at least one CD-associated *NOD2* variant compared to wildtype *NOD2* CD-patients (17.2% vs. 15.1%), but this difference was not significant. Similarly, the polymorphism Arg753Gln showed no significant interactions with *NOD2* variants in CD.

Our cohort has previously been genotyped for further IBD susceptibility variants. We next tested for possible interactions between the two *TLR2* polymorphisms and variants in *IL23R*, *ATG16L1*, *IBD5*, *TLR4* and *TLR9* [5, 28]. However, this test revealed no significant epistatic interactions for the polymorphisms in *TLR2* and known disease-associated variants in these genes.

We found a highly significant correlation between the number of GT_*n*_ repeats in intron 2 of the *TLR2* gene and the polymorphism Arg753Gln (rs5743708) (r = 0.0099038, *P* = 2.76 × 10^−10^) (see S1 Fig). This finding is in accordance with the previously reported strong linkage disequilibrium between the two polymorphisms [29]. A regional LD plot for the SNP rs5743708 (Arg753Gln) in *TLR2* on the Chr. 4q31.3 identified no other variant in strong LD (r^2^≥0.8) with this SNP (see S2 Fig). For the other three genes located in the same region of Chromosome 4: *KIAA0922* = *TMEM131L* (transmembrane protein 131-like), *RNF175* (ring finger protein 175) and *SFRP2* (secreted frizzled-related protein 2), no literature data linking them to inflammatory bowel disease or mycobacterial disease has been found.

## Discussion

In the present investigation we analysed the role of two functionally relevant polymorphisms in *TLR2*, the coding variant Arg753Gln (rs5743708) and the GT_*n*_ repeat microsatellite polymorphism in intron 2, in the susceptibility for IBD in a large European cohort. Both *TLR2* polymorphisms seem to affect immune responses (e.g. cytokine release) after stimulation with bacterial products [6, 7, 13, 14] and have previously been linked to susceptibility to mycobacterial disease [8, 17–20]. Given the considerable overlap between susceptibility for IBD and mycobacterial infection revealed by GWAS [1] and the substantial amount of still “hidden” heritability in IBD, the *TLR2* polymorphisms represent interesting candidates for CD and UC susceptibility.

Our study is the first to assess the distribution of the *TLR2* intron 2 GT_*n*_ repeat microsatellite polymorphism in IBD. Previous investigations reported an association of this polymorphism with various mycobacterial diseases such as nontuberculous mycobacterial lung disease [18], tuberculosis [19, 20] and also leprosy [13]. Overall, the number of GT_*n*_ repeats in our population varied between 13 and 28, with peak frequencies at 13, 19–21 and 24 repeats, which is in accordance with the distribution reported in the original description in Caucasians [7]. We observed a slightly higher frequency for short (S, with ≤ (GT)_16_) GT repeats in patients with CD and UC compared to controls. The genotype frequency for carriers of at least one S-allele was also slightly higher in IBD patients compared to controls. However, these differences were all not significant in the multivariate analysis. The further stratification of alleles with the cut-off of 18 GT repeats, which was found to best differentiate between patients and controls, did also not revealed any significant differences in the distribution in CD and UC compared to controls.

Because clinical phenotypes of IBD are partially genetically determined, we also conducted an extensive genotype-phenotype analysis to identify possible associations of the GT_*n*_ repeat microsatellite polymorphism with subgroups in CD or UC. This analysis found a slightly higher frequency of carriers of short GT_*n*_ repeats among the CD and UC patients with a need for immunosuppressive treatment, but this difference was also not significant in the multivariate analysis. Thus, our data do not provide evidence for a specific association of the microsatellite polymorphism with a phenotypic subgroup in CD or UC.

Besides leprosy, for which a clear link to CD susceptibility genes like *NOD2* [30] and *IL23R* [31] has been established, the microsatellite polymorphism in *TLR2* has been associated with further infectious conditions linked to CD-associated *NOD2* variants, like the susceptibility to develop spontaneous bacterial peritonitis in liver cirrhosis [15, 16]. Interestingly, in this setting the coexistence of longer GT_*n*_ repeats for the microsatellite polymorphism and *NOD2* mutations was associated to an additive risk to develop spontaneous bacterial peritonitis [16]. Our study instead, failed to show any interaction of the microsatellite polymorphism with *NOD2* variants in CD. Further epistasis testing did not reveal any interactions of the microsatellite polymorphism with other susceptibility IBD variants in *IL23R*, *ATG16L1*, *IBD5*, *TLR4* and *TLR9*.

Studies on the influence of the length of GT_*n*_ repeats on TLR2 function have shown higher promotor activity [7] and *TLR2* mRNA expression [13] as well as higher production of proinflammatory cytokines and lower production of anti-inflammatory cytokines [13, 14] for short GT_*n*_ repeats. Therefore, it has been speculated, that the shorter allele is much more prone to inflammation than mid-sized repeats and this would possibly explain why mid-sized alleles are most abundant in every race [7]. In comparison, S-alleles are relatively rare. As our study had sufficient power to detect disease associations for uncommon genetic variations with higher effect size, the negative results of the study exclude the GT_*n*_ microsatellite polymorphism as a disease associated variant with a significant effect size.

Regarding the low-prevalence variant Arg753Gln (rs5743708) in *TLR2*, a previous case-control association study comprising 285 European IBD patients (of which 106 had UC) described an association of this variant with pancolitis, with a relative risk of 3.3 in heterozygous patients [12]. In our well-powered investigation we found no significant association of this polymorphism with CD or UC but a comparable frequency of the polymorphism in all study groups. Recently Cheng et al. [32] performed an extensive meta-analysis on the association of *TLR2* and *TLR4* polymorphisms with IBD. The studies included in the meta-analysis assessed the frequency of the *TLR2* Arg753Gln polymorphism in a total of 718 patients with UC and 1454 patients with CD. The meta-analysis found no significant association of the polymorphism Arg753Gln with CD or UC in any of the genetic models [32] but the meta-analysis did not included a subgroup analysis of specific disease phenotypes in UC or CD. However, our subgroup analysis failed to show an association of the polymorphism with extensive disease in ulcerative colitis.

In conclusion our case-control association study revealed no significant role of the functional relevant polymorphisms in *TLR2*, the GT_*n*_ microsatellite repeat polymorphism in intron 2 and the Arg753Gln in the susceptibility to Crohn’s disease or ulcerative colitis.

## Supporting information

S1 Fig Correlation between the Arg753Gln (rs5743708) genotype and the number of GT_*n*_ repeats for the microsatellite polymorphism in *TLR2*. TLR2 753 mutated = carriers of at least one Arg753Gln allele.
(TIF)

S2 Fig Regional LD plot for rs5743708 in *TLR2* on Chromosome 4q31.3. The pairwise LD (r2) between this SNP and surrounding variants and the estimated recombination rate are plotted as a function of genomic position. The plot was constructed by SNAP (SNMP Annotation and Proxy Search, http://archive.broadinstitute.org/mpg/snap/ldplot.php) using the CEU population panel in the 1000 Genome Project (1000GP) Pilot 1 data and a 250 kilobases (kb) distance limit on each side. Three other genes are located in this region on Chromosome 4: *KIAA0922* = *TMEM131L* (transmembrane protein 131-like), *RNF175* (ring finger protein 175) and *SFRP2* (secreted frizzled-related protein 2).
(TIF)

S1 Table Frequencies for *TLR2* intron 2 microsatellite GT_*n*_ repeats genotypes in the study population. (DOCX)
