# Supplementary figures and images for: Functional Toll-Like Receptor (TLR)2 polymorphisms in the susceptibility to inflammatory bowel disease
Source: PLoS One. 2017 Apr 7;12(4):e0175180. doi: 10.1371/journal.pone.0175180 (PMC5384663; doi:10.1371/journal.pone.0175180)

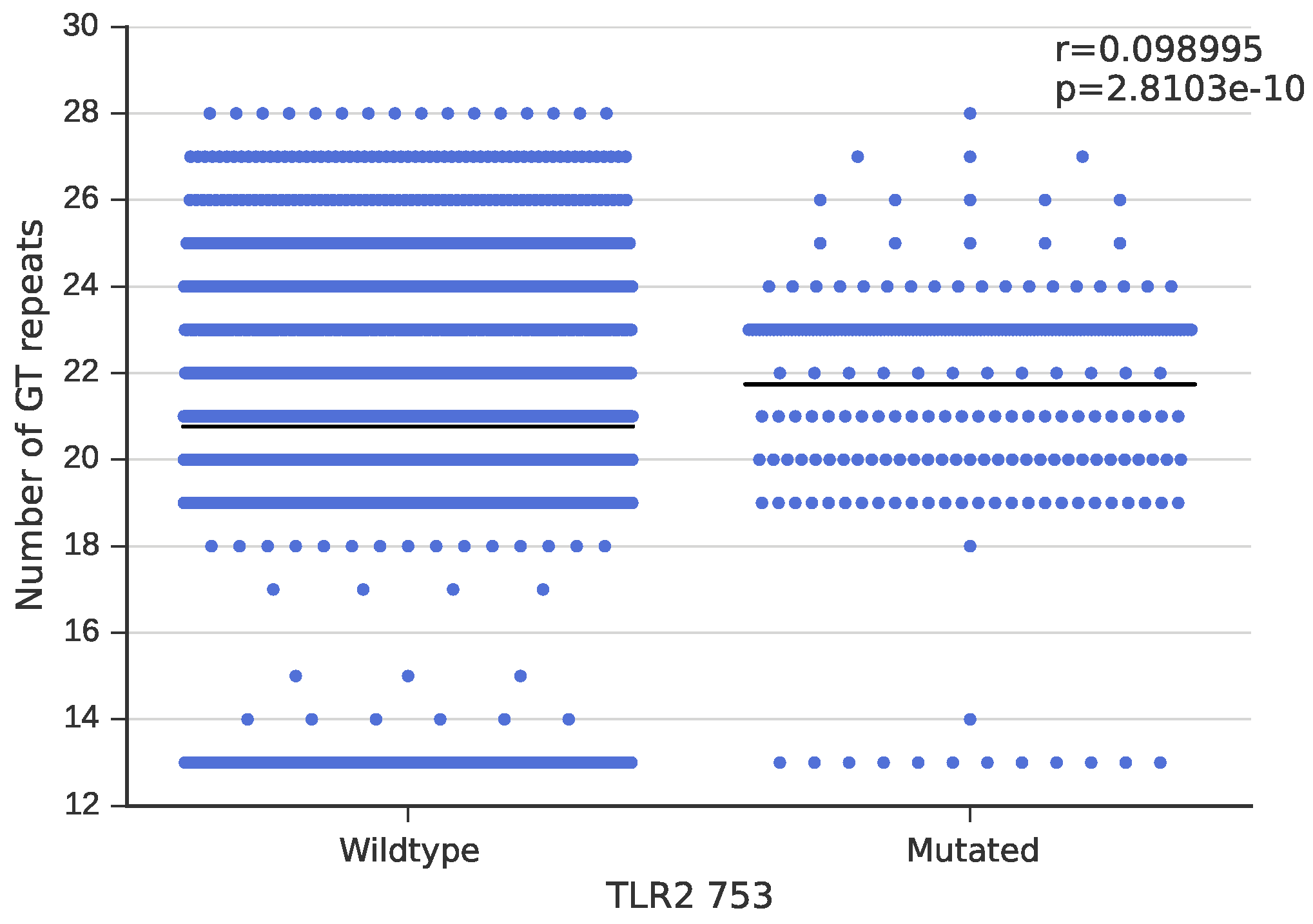

Supplement: S1 Fig — TLR2 753 mutated = carriers of at least one Arg753Gln allele. (TIF) [file pone.0175180.s001.tif]

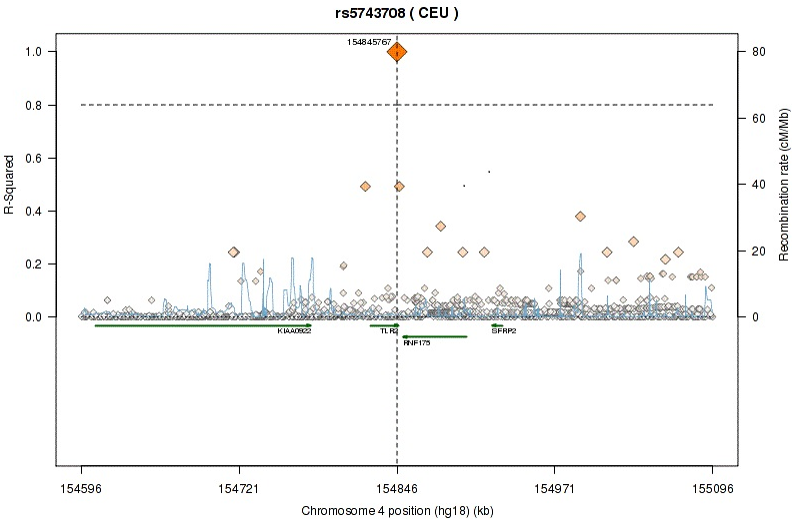

Supplement: S2 Fig — The pairwise LD (r2) between this SNP and surrounding variants and the estimated recombination rate are plotted as a function of genomic position. The plot was constructed by SNAP (SNMP Annotation and Proxy Search, http://archive.broadinstitute.org/mpg/snap/ldplot.php) using the CEU population panel in the 1000 Genome Project (1000GP) Pilot 1 data and a 250 kilobases (kb) distance limit on each side. Three other genes are located in this region on Chromosome 4: KIAA0922 = TMEM131L (transmembrane protein 131-like), RNF175 (ring finger protein 175) and SFRP2 (secreted frizzled-related protein 2). (TIF) [file pone.0175180.s002.tif]
