# Supplementary material for: Functional Toll-Like Receptor (TLR)2 polymorphisms in the susceptibility to inflammatory bowel disease
Source: PLoS One. 2017 Apr 7;12(4):e0175180. doi: 10.1371/journal.pone.0175180 (PMC5384663; doi:10.1371/journal.pone.0175180)
Supplement: S1 Table — (DOCX) [file pone.0175180.s003.docx]

**Supplementary Table 1.** Frequencies for *TLR2* intron 2 microsatellite GT*_n_* repeats genotypes in the study population

| **Genotypes**^2^ | **Crohn’s disease**  **N = 843** | **Ulcerative colitis**  **N = 426** | **IBD^1^**  **N=1269** | **Controls**  **N = 805** |
| --- | --- | --- | --- | --- |
|  | Frequency % | Frequency % | Frequency % | Frequency % |
| SS | 1.1 | 0.0 | 0.7 | 0.2 |
| SM | 10.8 | 12.0 | 11.2 | 8.9 |
| SL | 4.2 | 4.7 | 4.3 | 3.6 |
| MM | 38.6 | 39.0 | 38.7 | 40.1 |
| ML | 36.8 | 35.4 | 36.3 | 39.5 |
| LL | 8.7 | 8.9 | 8.7 | 7.6 |

^1^The category IBD (inflammatory bowel disease) represents the combined Crohn’s disease (CD) and ulcerative colitis (UC) cohort. ^2^S≤ (GT)_16_, (GT)_17_ < M < (GT)_22_ and L ≥ (GT)_23._
